# Spatial and temporal variations in seabird bycatch: Incidental bycatch in the Norwegian coastal gillnet-fishery

**DOI:** 10.1371/journal.pone.0212786

**Published:** 2019-03-13

**Authors:** Kim Magnus Bærum, Tycho Anker-Nilssen, Signe Christensen-Dalsgaard, Kirstin Fangel, Tom Williams, Jon Helge Vølstad

**Affiliations:** 1 Norwegian Institute for Nature Research (NINA), Lillehammer, Norway; 2 Norwegian Institute for Nature Research (NINA), Department of Terrestrial Ecology, Trondheim, Norway; 3 Institute of Marine Research, Bergen, Norway; Department of Agriculture and Water Resources, AUSTRALIA

## Abstract

The general decline of seabird populations worldwide raises large concerns. Although multiple factors are interacting to cause the observed trends, increased mortality from incidental bycatch in fisheries has proven to be important for many species. However, the bulk of published knowledge is derived from longline fisheries, whereas bycatch in gillnet fisheries is less studied and even overlooked in some areas. We present seabird bycatch data from a 10-year time-series of fishery data from the large fleet of small-vessels fishing with gillnets along the Norwegian coast—a large area and fishery with no prior estimates of seabird bycatch. In general, we document high rates of incidental bycatch (averaging 0.0023 seabirds/net, or approximately 0.08 seabirds/fishing trip). This results in an estimated annual bycatch between 1580 and 11500 (95% CI) birds in this fishery. There was a surprisingly high percentage (43%) of surface-feeding seabirds in the bycatch, with northern fulmar being the most common species. Among the diving seabirds caught, common guillemot was most numerous. Our findings suggest that coastal gillnet fisheries represent a more general threat to a wider range of seabird populations, as opposed to longline fisheries where surface-feeding seabird species seem to dominate the bycatch. The bycatch estimates for the Norwegian gillnet-fishery varied in time, between areas, and with fishing depth and distance from the coast, but we found no clear trends in relation to the type of gillnets used. The results enabled us to identify important spatio-temporal trends in the seabird bycatch, which can allow for the development and implementation of more specific mitigation measures. While specific time closures might be an efficient option to reduce bycatch for diving seabirds, measures such as gear modification and reduction in release of wastewater during fishing operation are probably a more effective mitigation approach for reducing bycatch of surface-feeding seabirds.

## Introduction

Incidental bycatch in gillnet fisheries has caused some of the highest recorded mortalities of seabirds worldwide, and a recent review estimated that each year a minimum of 400,000 birds die as a direct consequence of this type of fishery [1]. Increased mortality rates can potentially have serious effects on the population dynamics of seabird species as their life history strategies usually encompass long life spans and low annual reproductive output (e.g., [2]). This is especially concerning as the majority of seabird populations around the globe are in decline [3], with the conservation status of many seabird species listed as highly threatened [4]. However, the actual effect of the mortality from bycatch on seabird populations is usually unknown, although circumstantial evidence of a large general effect exists [5]. The apparent lack of population-level effects of gillnet bycatch reported in the literature may be a consequence of multiple factors, but it seems that the gillnet fishery has largely been overlooked as a threat to seabird populations [6]. Consequently, there is a large degree of uncertainty with respect to how seabirds are killed as incidental bycatch in many such fisheries [6, 7], and the current knowledge is based on short-term studies that are highly fragmented in space and time.

A great range of factors are likely driving the general decline of seabirds, including direct and indirect effects of climate change [8], commercial fisheries [9], introduced mammals [10], pollution and coastal development [4]. Many of these factors might also act in synergy to intensify the negative effect on the populations [11], which complicates management efforts to counteract the declines. Nevertheless, bycatch-induced mortality of seabirds is probably among the most manageable problems in the short term. Thus, it is important to get a good understanding of the extent and variation of incidental bycatch in the different fisheries. The research on incidental bycatch of seabirds has primarily focused on longline fisheries (e.g., [12–14]). This is also the case for much of the research on effective mitigation measures to reduce the bycatch of seabirds (e.g., [7, 15, 16]). There are, however, obvious gaps in our knowledge of where, how and why bycatch of seabirds in gillnet fisheries occurs, what the possible mitigation measures of such bycatch are (but see [17–19]), and to what extent it affects the seabird populations.

Analyses that aim to integrate multiple potential factors in a spatio-temporal perspective to explore seabird bycatch require rather large datasets, which probably explains why such analyses are scarce in the literature. Therefore, deriving more knowledge from long-term data series and larger scales is essential to provide better estimates of the number of seabirds killed, spatio-temporal trends in bycatch rates and population-level impacts. This is necessary to increase our understanding of the susceptibility of species and, eventually, identify effective mitigation measures.

In this study, we utilized a comprehensive time series dataset of complete catch records, involving a total of 43 fishing vessels over a 10-year period, to investigate and estimate rates of incidental bycatch of seabirds in the Norwegian coastal gillnet fishery. The sampled fishing fleet represents a large-scale fishery that spans the entire Norwegian coast (58–71°N) and is fundamentally similar to other small-boat gillnet fisheries around the globe. These are fisheries that in general are widespread and common. The main goal of the study was to contribute to the global knowledge base on seabird bycatch by 1) presenting bycatch rates from areas and fishing equipment with few prior observations; 2) exploring spatio-temporal patterns of the bycatch, and the extent to which these patterns were associated with the type and use of fishing equipment; and 3) assessing the value of the results in a wider, international context. To achieve this, we also explored the variation in seabird bycatch in relation to foraging characteristics of seabirds (i.e., diving and surface-feeding) to uncover possible differences between the two groups. Such information is crucial to uncover which populations of seabirds are most likely to be affected by this type of fishery and identify potential ways forward to reduce the bycatch.

## Materials and methods

In this study, we utilized data from the Norwegian Reference Fleet, a group of Norwegian fishing vessels contracted by the Institute of Marine Research (IMR) to provide detailed information about their fishing activity and catches, including bycatch of marine mammals and seabirds, through trained self-sampling on a regular basis (read more on www.imr.no/temasider/referanseflaten/en). Each of these vessels reports complete, detailed information on the composition of catches from fishing operations sampled systematically through time. From each sampled fishing operation, the catch of all fish is recorded in numbers and weight by species. Bycatch of seabirds and marine mammals are reported in numbers by species. In this sampling program, vessels are stratified according to gear and ICES statistical area (see description at http://www.ices.dk/marine-data/maps/Pages/ICES-statistical-rectangles.aspx). As the reference fleet comprises a variety of vessels, and we wanted to explore trends representative for the coastal small-boat fisheries, we only used gillnet data from the coastal part of the fleet. For the years used in the study (2006–2015), the coastal part of the fleet was limited to include 9–15 m long vessels fishing along the coast of Norway, with one additional 21m long vessel included in the fleet from 2009–2012. There were no specific permits or permissions required for the sampling.

A trained research technician from the IMR is the prime contact and serves as mentor for the fishers on each vessel in the reference fleet. The mentor is responsible for providing training and support, and for quality control and quality assurance of data and biological samples delivered to the IMR. The training of fishers includes regular visits to every reference fleet vessel at sea by IMR scientific staff, annual meetings between IMR and reference fleet participants, subject-specific workshops and training in taxonomy. The data and samples sent monthly to the IMR are first checked by the mentor and then independently by a technician, before the data are entered into the IMR’s research database. The data are further checked using a test program that identifies anomalies, and then scrutinized manually for possible errors, bias and deviation from the protocol. The IMR has an agreement with the fishing authorities that the data from the vessels in the reference fleet program will not be used directly for enforcement of restrictions or sanctions. This is in order to mitigate against vessels changing their behaviour that could cause bias in the self-reported data that are applied to the fishing fleet in general. Concerns about the potential bias in the data are further mitigated by the training given by the mentor in order to improve the fishers’ understanding of why it is important to not change their fishing behaviour and to follow the protocol for catch reporting. Moreover, the regular occurrence of bycatches of seabirds and marine mammals in the catch reports from the reference fleet demonstrates that self-reporting of seabird bycatches is possible for this fishery.

To explore the extent and variability of seabird bycatch in gillnet fisheries along the Norwegian coast, we applied two main approaches: 1) A stratified mean estimator and 2) a generalized mixed model (GLMM); both approaches described in more detail below. The Norwegian Directorate of Fisheries registers catches from all fishing trips made in the entire Norwegian coastal fishing fleet (i.e., all vessels that participate in commercial fishing). However, as only a few parameters other than the number of trips (trip effort), area and catch are recorded, the stratified mean estimator is intended as a rough estimator for predicting seabird bycatch for the whole fleet to allow comparisons with that of other fisheries throughout the world. The GLMM approach aimed to describe which parameters might affect seabird bycatch rates the most, detect spatio-temporal patterns and to provide better guidance on how to construct effective mitigation measures. It was also set up to better account for the non-normal and possible zero-inflated distribution of seabird bycatch data.

### Data

**Fig 1:**
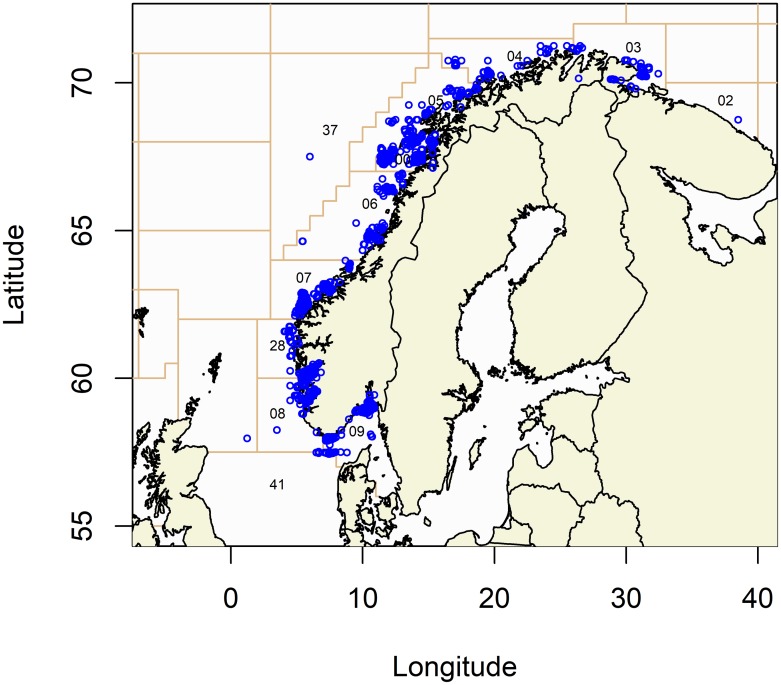
Fishing trips used for bycatch estimation. Map of the Norwegian coastline with registered trips (open blue circles) for the coastal reference fleet utilizing gillnets during 2006–2015. Numbers refer to statistical fishing area, where 2, 3 and 4 are referred to as north-east, 0, 5 and 37 are referred to as north-west, 6 and 7 as middle and 28, 8, 9, and 41 as south in the analysis.

The analysis focused on bycatch data provided by the coastal reference fleet from the years 2006–2015 for fishing operations using gillnets designed to fish close to the sea bottom. However these nets are also set closer to the surface with the use of buoys. This totalled 15,894 net-fishing trips, conducted by 43 different vessels (see spatial coverage for the fishing trips in Fig 1). An average fishing trip in this dataset involved the setting of 36 (SD ± 26) nets. Unfortunately, we did not obtain exact information on soak time. A total of 1191 seabirds were registered as incidental bycatch (see Table 1 for annual summaries). Due to missing data for some variables of interest (e.g., exact UTM coordinates absent for some registrations), a subset of 13,980 trips with 1080 seabirds recorded as incidental bycatch were utilized in the analytical modelling. The omitted data points did not represent any obvious patterns concerning the variables of interest or bycatch counts.

**Table 1:** Total numbers of trips, vessels and seabird bycatch.

Year	Number of trips	Number of vessels	Seabird bycatch
2006	1797 (91946)	20 (3618)	70
2007	1600 (92484)	16 (3568)	31
2008	1300 (91416)	17 (3744)	299
2009	1664 (76383)	18 (3378)	127
2010	1798 (83457)	20 (3118)	121
2011	1805 (79874)	20 (3167)	198
2012	1372 (76995)	21 (3121)	80
2013	1770 (68115)	19 (2808)	154
2014	1278 (68923)	15 (2629)	79
2015	1510 (61059)	19 (2526)	32

Total number of trips, vessels and incidental seabird bycatch recorded for each year by the coastal reference fleet. Numbers in parentheses correspond to landings/trips for the complete coastal fishery fleet registered by the Norwegian directorate of fisheries. The numbers only portray trips where gillnets/set nets were used as primary fishing equipment (i.e., excluding floating nets).

For the same years (2006–2015), the Norwegian Directorate of Fisheries provided mandatory fish catch records from 790,652 net-fishing trips conducted by a total of 7720 vessels along the Norwegian coast, for which the data extracted from the reference fleet (i.e. trips with vessels of length 9–21 m) were considered representative (for details see Table 1). We used these trips to expand seabird bycatch estimates calculated from the reference fleet data to the fishery as a whole.

### Seabird bycatch estimates: Stratified mean estimator

Bycatch rate was estimated as a stratified mean bycatch per trip (S1 Equations), based on data from the coastal reference fleet. The estimated bycatch rate per trip was extrapolated to the total number of trips by all representative vessels registered by the Norwegian Directorate of Fisheries. To obtain a more comparable estimator to other estimators in the literature, we also calculated seabird bycatch per unit effort (BPUE) as a stratified ratio estimator, specifically as the mean ratio of bycatch in number of birds per net in the reference fleet. However, as the official Norwegian fishery statistics has no overall information on the total number of nets set, we could not use this estimator to project to the total numbers of seabirds caught as incidental bycatch for the whole fleet.

### Seabird bycatch estimates: GLMM

We explored the variation in seabird bycatch using a GLMM framework in the statistical software R, version 3.4.3 [20]. As we anticipated bycatch to vary with the feeding behaviour of seabirds (i.e., diving or surface-feeding, see distinctions of species into feeding type in Table 2), we fitted two sets of models using the bycatch numbers of the two specific feeding types as responses. Specifically, we utilized the *glmmADMB* package [21, 22] to fit different candidate models to describe possible trends in the bycatch for diving seabirds and surface-feeding seabirds. The response variable was numbers of seabirds caught per fishing trip, encompassing relatively few trips with bycatch and many trips without bycatch (i.e., many zero-observations). Data exploration prior to model fitting revealed three extreme bycatch events consisting of trips with 52, 52 and 83 seabirds caught. All three incidents happened in the same area (northeast), year (2008), and month (March/April) for a single species (the common guillemot *Uria aalge*), and involved only one specific vessel on three consecutive trips. The nets on these trips were set at depths between 16 and 20 m, and relatively close to shore. As these incidents were limited to a small local area and seemed exceptional within the 10-year time-series in terms of number of seabirds caught, we decided to exclude them from the GLMM analysis. The rationale behind this decision was that it would be virtually impossible to get statistical reliable predictions from these very rare and extreme events in the data.

**Table 2:** Representation of seabirds in catch.

Type of feeding behaviour	% of total	Species/family	% of total
		Razorbill *Alca torda*	8.4
		Common guillemot *Uria aalge*	29.3
		Common eider *Somateria mollissima*	1.4
Diving	57.1	Cormorants *Phalacrocorax* spp.	4.3
		Atlantic puffin *Fratercula arctica*	3.2
		Black guillemot *Cepphus grylle*	1.4
		Northern gannet *Morus bassanus*	9.0
		Northern fulmar *Fulmarus glacialis*	32.7
Surface-feeding	42.9	Black-legged kittiwake *Rissa tridactyla*	6.1
		Gulls *Laridae* spp.	4.1

Percentage of seabird species represented in the catch of the small-vessel costal reference fleet from 2006 to 2015. The percentages are based on the full dataset, including three extreme bycatch events consisting of trips with 52, 52 and 83 seabirds caught. The birds were divided based on taxon (species/family) and foraging behaviour: diving (grey) and surface-feeding (white). Total numbers of seabirds caught by the reference fleet in the period amounted to 1189 seabirds.

For the models, we considered response distributions such as Poisson, negative binomial, zero-inflated Poisson and zero-inflated negative binomial distributions. We considered the negative binomial distribution and zero inflation to allow for overdispersion and an excess of zeros due to patchiness in occurrence of seabirds and bycatch. To assess the most appropriate distribution and model, we first tested and graphed the response, following Friendly and Myer [23], to determine whether the negative binomial distribution was more appropriate than the Poisson distribution. This procedure favoured a negative binomial GLMM for both surface-feeding and diving seabirds. We then constructed 14 candidate models utilizing the variables shown in Table 3 as possible fixed effects, and *Vessel ID* as a random intercept in all candidate models. The models were similar for both diving and surface-feeding birds.

**Table 3:** Variable descriptions.

Variable	Description
Year (y)	Year of specific fishing trip.
Month (m)	Month of specific fishing trip.
Net mesh size (ns)	Grouped into five categories: 1) 35–85 mm, 2) 86–135 mm, 3) 136–185 mm, 4) 186–235 mm, 5) unspecified.
Distance from coast (dc)	Distance to nearest shoreline. Calculated based on GPS positions and map polygons.
Fishing depth (fd)	Shallowest fishing depth for the specific fishing nets. Not necessarily related to depth of the sea-bottom.
Area (a)	Statistical fishing area, divided in four main groups (northeast, northwest, middle and south, see Fig 1).
Number of nets set (nn)	Number of nets set at the specific fishing trip.
Vessel ID	Registration code, specific for each vessel in the fishing fleet.

Description of available candidate variables utilized for exploring variation in seabird bycatch in the Norwegian coastal gillnet fishery.

Data exploration prior to fitting the models indicated both temporal (variation between months and years) and spatial variation (variations between areas) in the seabird bycatch; variables dealing with time and area were thus included in most candidate models. As we also expected that the number of nets set would affect the number of seabirds caught, and that this effect would vary with fishing depth (e.g., probably no effect at depths too deep for diving seabirds), we included an interaction between fishing depth and number of nets in most candidate models. Further, we explored effects of fishing equipment and distance to shore, where the effect of fishing equipment (i.e., mesh size) was included only in the most supported models following a prior model selection on models 1–13 (see all candidate models in Table 4). We considered both fishing depth and distance to shore in the same models, as they were not very related (R^2^ = 0.03, F_1,13975_ = 435.2, p < 0.001). We included interacting effects that were biologically feasible, but restricted this to two-way interactions for parsimony. Specifically, we explored how the effect of distance to shore and fishing depth change in the spatio-temporal environment. Preliminary analyses showed no obvious bycatch trends in relation to the abundance of specific fish species in the catches. Therefore, we did not include information regarding target species for the fisheries in the models. Further, we believe the variables explored (Table 3) gave a more general description of a fishery, rather than a fishery defined by target species.

**Table 4:** Candidate models with AIC-values.

Model / Fixed effect structure	df	ΔAIC_Diving_	ΔAIC_Surface_
Effects of number of nets and fishing depth			
*1*. *No fixed effect*	3	118	53.4
*2*. *~ nn x fd*	6	67.7	45.7
*3*. *~ nn + fd*		67.8	44.5
Effects of temporal and spatial variables			
*4*. *~ y + m + a*		65.4	12.8
*5*. *~ y + m + a + nn x fd*	23	17.8	54.3
*6*. *~ y + m + a + nn x fd + dc*	24	17.9	11
*7*. *y + m + a + dc + nn + fd*		19.9	9
***8*. *~ y + m + a x dc + nn x fd***	**27**	**0**	1.5
*9*. *~ y + m x dc + a + nn x fd*	29	25.1	6.1
*10*. *~ y + m + a + nn x fd + fd x dc*	25	19.7	12.2
*11*. *~ y + m + a x fd + nn x fd*	26	14.5	14
*12*. *~ y + m + a x fd + nn x fd + dc*	27	13.8	12.3
*13*. *~ y + m + a + nn + dc*	22	57.5	8.3
*14*. *~ y + m + a x fd + dc*	25	18.3	11.3
*15*. *~ y + m + a x dc + fd*	25	3.9	0.7
***16*. *~ y + m + a x dc***	**21**	41.5	**0**
*17*. *~ y + m + a + dc*		67.2	**9.3**
Effects of type of fishing gear (response specific)			
*18*. *~ y + m + a x dc + nn x fd +ns*	31	3.7	
*19*. *~ y + m + a x dc + ns*	29		2.4

Candidate models for exploring variation in seabird bycatch in the Norwegian coastal net fishery, including degrees of freedom, *ΔAIC* as calculated from *bbmle* package [25]. *ΔAIC*_*Diving*_ and *ΔAIC*_*Surface*_ are *ΔAIC* values from the model selection for diving and surface-feeding seabirds, respectively. Variable description used in the fixed effect structure can be seen in Table 3. All candidate models were fitted as negative binomial GLMM’s, with *Vessel ID* as a random intercept.

The most supported models were selected based on Akaike information criterion (AIC) values [24], where the highest ranked model, assessed with the *bbmle* package [25], was chosen for further analysis. We also assessed models with considerable support (i.e., within two *ΔAIC* of the most supported model) besides the highest ranked model when applicable. We then compared the most supported model with its zero-inflated equal (i.e., the same model with zero-inflated negative binomial distributions), and also a different parameterization of the negative binomial model (var(Y_*ij*_) = ϕμ), using *AIC* values to assess the different models. We validated the final model by plotting the Pearson residuals against the fitted values and each covariate. As part of the model validation, we also checked the Pearson residuals for temporal and spatial independence using the *gstat* package version 1.1–3 [26], following Zuur et al. [27]. We found no clear signs of temporal or spatial correlations. For the most supported models, a post hoc Markov chain Monte Carlo (MCMC) fit, using the *glmmADMB* package, was run to further assess parameter variability and effective size (i.e., the number of samples corrected for autocorrelation).

## Results

At the fishing-trip level, bycatch of seabirds in our study system was relatively rare with only 2% of the trips reported to have any such bycatch, and approximately 85% of the bycatch events involved less than five seabirds caught per trip. The remaining 15% of the events involved up to 29 seabirds per trip, and up to 83 seabirds per trip when including the three extreme events. In total, there was a somewhat surprisingly even distribution of diving (57%) and surface-feeding seabirds (43%) in the net catches, with northern fulmar (*Fulmarus glacialis*, hereafter fulmar) and common guillemot dominating the catches (Table 2).

### Seabird bycatch estimates: Stratified mean estimator

**Fig 2:**
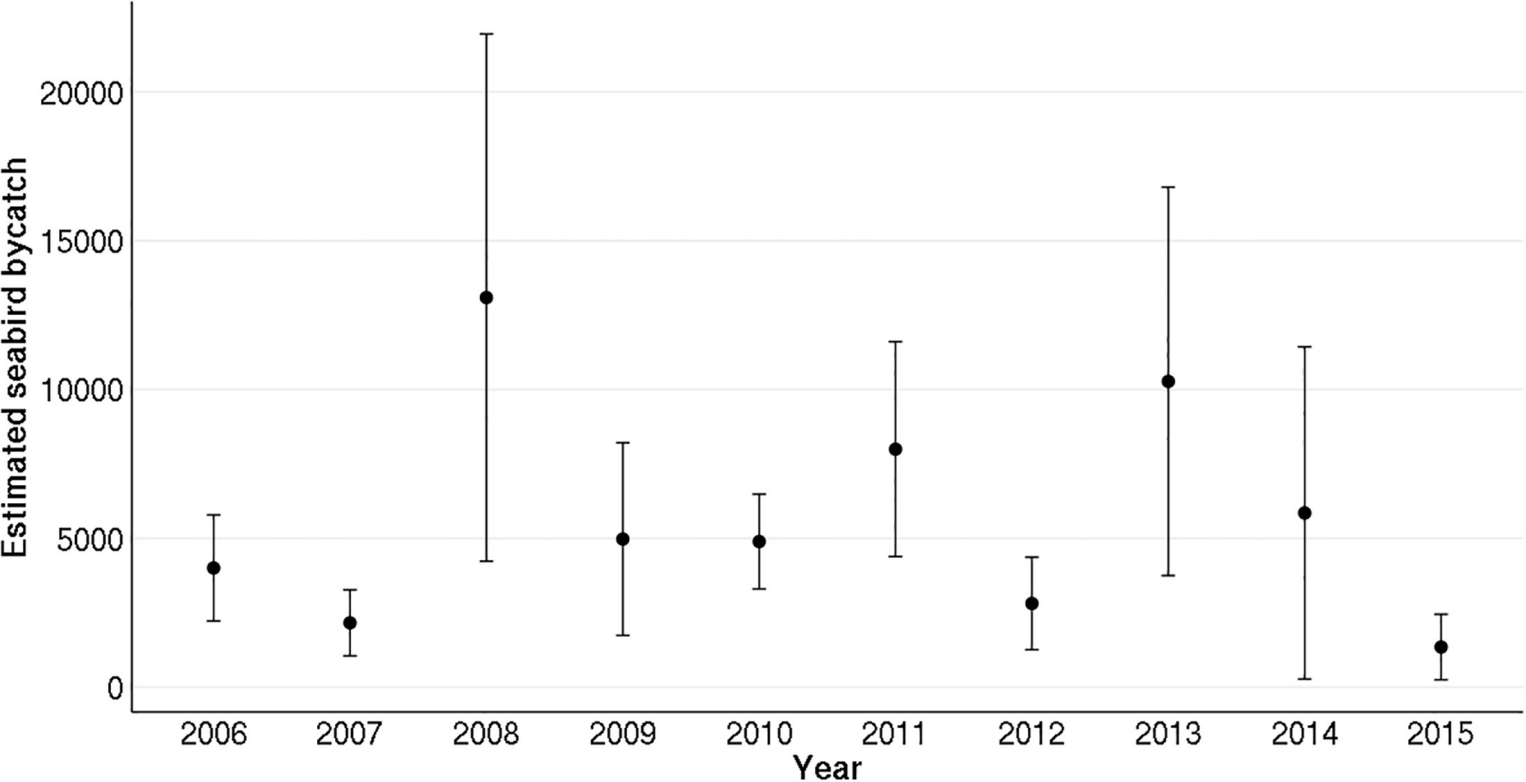
Point estimates of seabird bycatch. Point estimates (± SE) of yearly incidental bycatch of seabird from 2006 to 2015 in the Norwegian small-vessel gillnet fishery. The estimates are based on catches from the reference fleet, excluding three extreme bycatch events in 2008, and scaled to all trips registered within the representative fishery.

Mean estimated seabird bycatch per trip was calculated to ≈ 0.08 (SE = 0.03) seabirds across all years using the complete dataset. Excluding the three extreme bycatch events in 2008 reduced the mean to ≈ 0.06 (SE = 0.02). However, estimated bycatch per trip still varied considerably between years, with no clear trend. When including the extreme bycatch events, the estimated bycatch for the complete coastal Norwegian net-fishery fleet from 2006–2015 amounted to approximately 65,220 (SE = 24,702) seabirds. Excluding the three extreme events reduced the estimate to 52,019 (SE = 14,918) individuals (cf. Fig 2 for all annual estimates). The variation between estimated yearly bycatch for the whole coastal Norwegian net-fishery fleet followed the variation between estimated bycatch rates closely, as fishing effort in the fishery varied little between years.

As a more fine-scaled measure of bycatch for the reference fleet, the stratified ratio estimator was calculated to ≈ 0.0023 (SE ≈ 0.001) seabirds per net using the whole dataset, and ≈ 0.0019 (SE ≈ 0.0006) seabirds per net when excluding the three extreme bycatch events.

### Seabird bycatch estimates: GLMM

The model selection procedure revealed a different fixed effect structure for diving and surface-feeding seabirds, suggesting different drivers behind the bycatch events. For both feeding types, the most supported models were negative binomial GLMMs.

**Fig 3:**
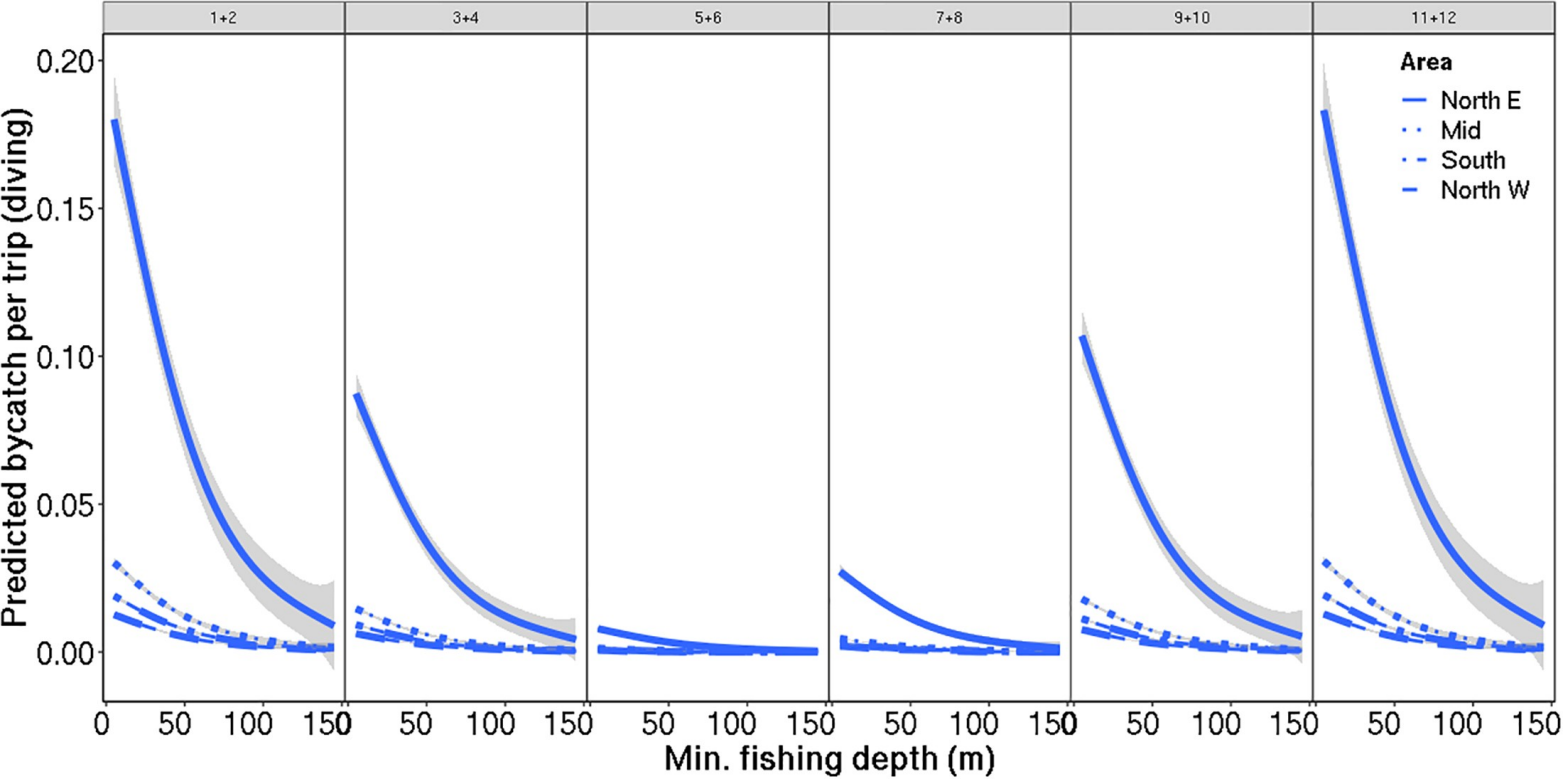
Predicted bycatch of diving seabirds: Fishing depth. Predicted bycatch of diving seabirds per fishing trip (± SE indicated in grey) against minimum fishing depth, divided by area (type of lines) and months (panes). The predictions are averaged over all fishing trips, years and distances to coast. Further, the predictions assume the use of 36 fishing nets per trip, which is approximately the mean number of nets used per trip.

**Fig 4:**
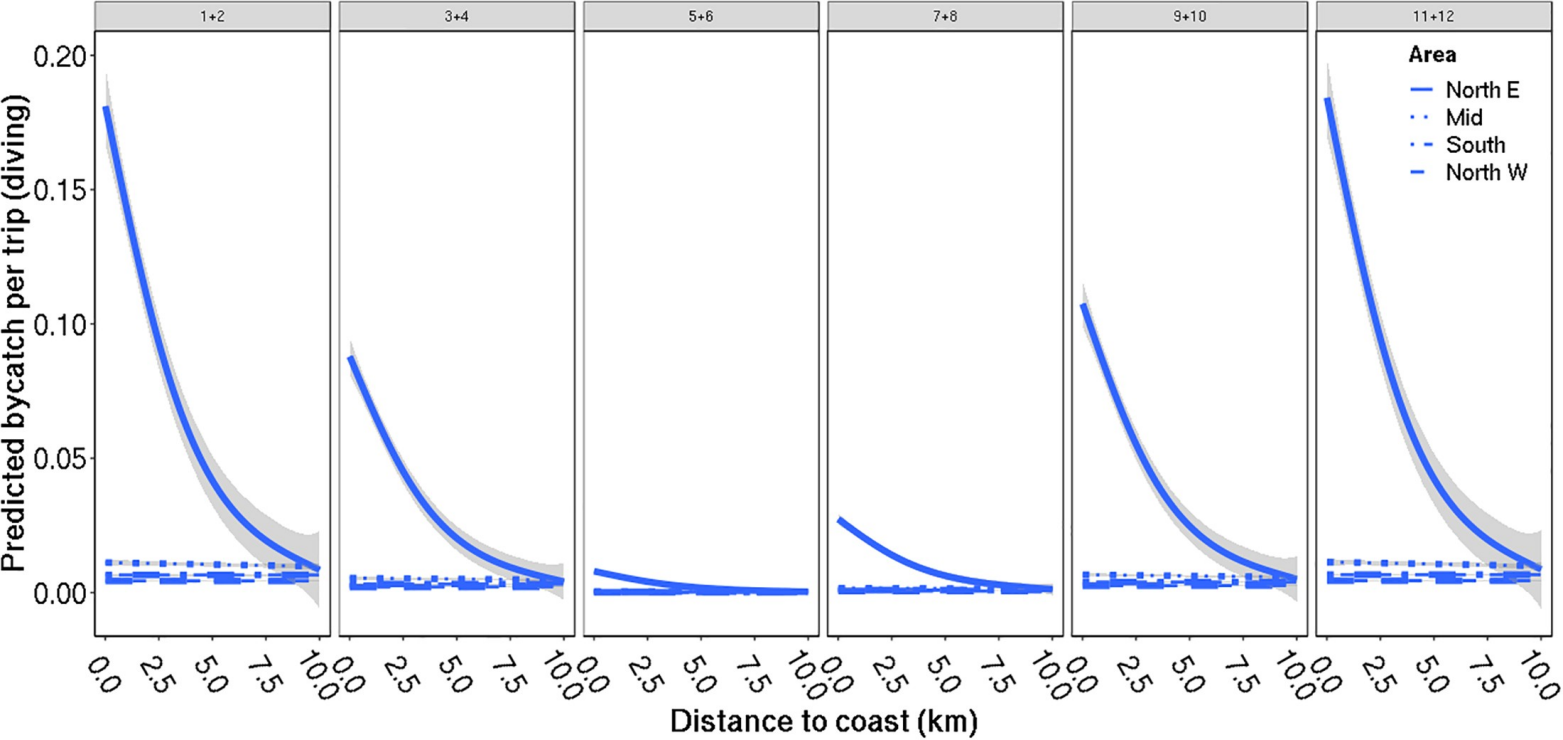
Predicted bycatch of diving seabirds: Distance to coast. Predicted bycatch of diving seabirds per fishing trip (± 95% CI indicated in grey) against distance to coast, divided by area (colour of lines) and months (panes). The predictions are smoothed and averaged over all fishing trips, years, and for minimum fishing depth from 5 m to 100 m. Further, the predictions assume the use of 36 fishing nets per trip, which is approximately the mean number of nets used per trip.

For diving seabirds, one model got substantially more support (based on AIC) than the alternative candidates. The fixed effect structure for this model included year, month, area, distance from coast, number of nets set and fishing depth as fixed effects, where the effect of distance to coast varied with area and the effect of number of nets set varied with fishing depth (Table 4). The temporal trend revealed increased bycatch rates in winter, with highest predicted bycatch between November and January, while the bycatch rates were lowest in May and June (Fig 3). The bycatch rate was higher in northeastern Norway compared to the other areas, in particular close to shore (Fig 3) and at minimum fishing depth (Fig 4). All areas had elevated bycatch rates of diving seabirds at minimum fishing depth. The effect of distance to shore was only apparent in northeastern Norway, where it was very similar to the effect of minimum fishing depth, indicating co-occurring events of high bycatch rates both close to shore and with nets being set at a shallow fishing depth.

**Fig 5:**
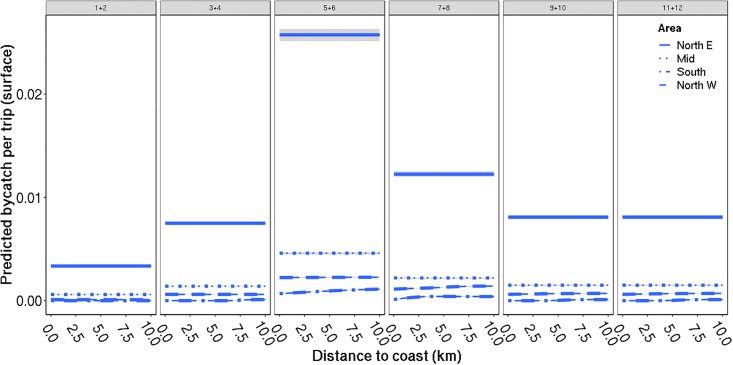
Predicted bycatch of surface-feeding seabirds: Distance to coast. Predicted bycatch of surface-feeding seabirds per fishing trip (± SE indicated in grey) against distance to coast, divided by area (colour of lines) and month (panes). The predictions are averaged over all fishing trips and years. Further, the predictions assume the use of 36 fishing nets per trip, which is approximately the mean number of nets used per trip.

For surface-feeding seabirds, three models had considerable support (Table 4). The highest ranked model included year, month, area and distance to coast as fixed effects, with the effect of distance to coast varying with area. The second- and third-most supported models also included an effect of fishing depth either as an additive effect (second-most supported model), or as an interactive effect with number of nets set (third-most supported model). However, as the apparent effect of fishing depth for surface-feeding seabirds seemed unreasonable, with elevated bycatch at increasing fishing depth, we chose to focus on the predictions from the most supported model only. The temporal bycatch trend was opposite to that of the diving seabirds, showing a general peak in May and June, with declining bycatch rates towards and during the winter months (Fig 5). Also, for surface-feeding seabirds, bycatch rates were generally higher in northeastern Norway. The apparent effect of distance to shore varied from area to area, from a small tendency of increasing bycatch rates with increased distance to the shore in the south, central and northwest areas to seemingly no effect of distance in the northeastern area (Fig 5). It is also interesting to note that our most supported model for surface-feeding seabirds seemed to underestimate the bycatch rate in general and did not explain the variation in the data as well as for the model for diving birds. Thus, although the model for surface-feeding seabirds still showed obvious trends in the bycatch, we probably lacked some unmeasured variables related to the variation in surface feeding seabirds, particularly with regards to describing events where multiple seabirds were taken.

## Discussion

In this study, we present estimates and distinct spatio-temporal patterns of seabird bycatch in the extensive small-vessel gillnet fishery along the entire Norwegian coast. Our findings represent new and important contributions to the seabird bycatch literature, especially because statistically-valid knowledge of the extent of bycatch for this large-scale type of fishery has been very limited. Even when excluding extreme events, the associated total estimate of bycatch in the 10-year study period amounted to approximately 52,000 seabirds (SE = 15,000), although with large variation among years. Approximately 60% of this bycatch consisted of equal numbers of common guillemots and fulmars, which amounts to an approximate estimate of 1300 birds of each of the two species being killed annually as bycatch. The high percentage of surface-feeding seabirds taken was perhaps the most surprising result from our findings and contradicts the general perception of surface-feeding birds not being as susceptible to bycatch in bottom-set gillnets as diving species [1] (but see [28]). These findings are thus intriguing and concerning from an international perspective. Many surface-feeding procellariform species have been identified as threatened by incidental bycatch in longline fisheries [7]. Considering the world-wide use of gillnets, our results make it reasonable to question if surface-feeding seabirds are under-represented in the international literature on bycatch in gillnet fisheries.

The temporal trend in bycatch, with higher bycatch rates of diving species in winter than in summer, could be expected for several reasons. One is that most of these birds, comprising 41% of the bycatch, were of species that only breed in a relatively small number of colonies, many of which are within protected areas where fishing close to land is restricted between 15 April and 31 July. In comparison, only 7% of the bycatch belonged to more wide-spread diving species (common eider, cormorants and black guillemot). Also, the five most abundant species, constituting 85.5% of the bycatch, are highly pelagic species that typically forage on food supplies found far offshore where gillnet fisheries are less common. Outside the breeding season, seabirds are free to move more opportunistically. Further, the pelagic species are sometimes attracted to forage in coastal areas because of temporal availability of food, such as capelin *Mallotus villosus* (that spawns mainly on the Finnmark coast), or larger herring *Clupea harengus* brought to surface by whales. The fisheries targeting such fish stocks, or targeting larger predatory fish attracted to the stocks (such as cod *Gadus morhua*), may also represent an attractive source of food, especially in periods where natural food is less available because of prolonged periods of bad weather and reduced light conditions.

The peak in bycatch of surface-feeding seabirds, especially fulmars, in May and June is somewhat more puzzling. However, most seabirds are still incubating early in the breeding season, leaving the off duty parent more time to explore larger areas than when both adults are caring for the offspring later in summer. It has also been shown that the proportions of immature fulmars in the bycatch is higher in this period than later in the season [29], possibly because these birds are likely to prospect for potential future colonies more when the established breeders are busy caring for their young.

Our results provide new information for improvement of mitigation methods to reduce and avoid seabird bycatch in gillnets, and also address the urgent need to understand which species groups are susceptible to bycatch in such fisheries in order to initiate the most effective mitigation measures. For diving seabirds, the clear trend of elevated bycatch rates in specific areas, at specific fishing depths, distances to shore and time periods, indicates that measures such as space/time closures in the fishery might be an effective mitigation action for reducing seabird mortality. In particular, ensuring a minimum fishing depth and minimum distance from the coast for the fishery in the winter months may have the potential to lower the bycatch rate for diving seabirds in general. Another option would be to enforce obligatory gear change or modifications (e.g., [30, 31]). By enforcing specific closures or obligatory gear modifications, for a shorter time, the mitigation measures might effectively be balanced against associated social benefits of the fisheries, such as for example food security and local job opportunities.

For surface-feeding seabirds, we also recognized a distinct spatio-temporal pattern, with the highest bycatch rates in May and June, and generally higher bycatch rates in the northeast and central fishing areas. The apparent small increase in bycatch rates for some areas, as well as the effect of increased bycatch rates with minimum fishing depth as shown by our second- and third-most supported models, are more difficult to explain from a biological perspective. It is possible, however, that both distance to the coast and minimum fishing depth are associated with variables not included in our analysis. One could, for example, expect distance from the coast to be associated with increased wave height, or rougher seas in general. Thus, the observed tendency of increased bycatch with distance to the coast could simply be due to more movements and/or decreased visibility of the nets while they are being set or hauled, increasing the risk for birds to get entangled. Also, the effect of distance from the coast might be related to the distribution of the susceptible species in Norwegian waters, with fulmars feeding further away from the coastline than other surface-feeding seabirds, as the species is overrepresented in the catch. Further, as there was no clear effect of minimum fishing depth, we suspect that most surface-feeding seabirds are stuck in the nets during setting or hauling. This hypothesis was supported by the fishers that collected the data, when questioned on the specific patterns of this bycatch. In their experience, the movement of the boat and fishing gear when setting and hauling in rough conditions most often lead to birds getting trapped in the nets. During fishing, nets are often hauled and set again in one operation. The associated presence of fish on the surface attracts surface-feeding birds, which risk being dragged down quickly if they are in contact with the fishing gear when set again. An understanding of how and why these birds are caught points to some obvious actions that can likely help reduce the bycatch of surface-feeding seabirds. Measures such as scaring lines, gear modifications and reduction in release of wastewater during fishing operation (e.g., [16, 32]) are probably a more effective mitigation approach for this group of birds than the implementation of space/time closures. For both surface feeding and diving seabirds, we did not find a clear effect of mesh size. Thus, we do not find any support for changing mesh size as a possible way of mitigating seabird bycatch.

On a population level, the effects of bycatch mortality depend not only on the number of birds caught, but also on the sex and age structure of the birds killed, which source population they belong to, and the status of those populations [33, 34]. In our study, we did not have access to this information. Previous bycatch studies of fulmars in the same areas have, however, shown that adult male birds dominated the bycatch and that these birds likely originated from populations breeding in more temperate regions [35]. It is therefore plausible that the same applies in this study. Fulmars travel far and have extensive foraging ranges [36] and birds on the Norwegian coast could thus originate from colonies far away from the point of capture. An estimated 2.4–4.4 million pairs of fulmars breed in the northeastern Atlantic [37, 38], but several of the populations are now in decline [39, 40], making it imperative to identify the drivers of the negative trend. This especially applies to the small and nationally endangered breeding population of fulmars along the Norwegian mainland (e.g., [41]).

Common guillemots, the second most affected species in the study, were primarily taken as bycatch during the winter in northeastern Norway. Preliminary results from the SEATRACK project, which tracks seabirds over time (http://www.seapop.no/en/seatrack/), show that this is a key wintering area for common guillemots from colonies in the southwestern Barents Sea (http://seatrack.seapop.no/map/), whereas birds from colonies further south rarely stay in the Barents Sea in winter. The Finnmark and Bjørnøya populations in Norway have increased substantially since a huge population crash in the mid-1980s [41, 42], and now probably total more than 300,000 breeding birds. It is difficult to assess what effect the bycatch of 1300+ common guillemots per year has had on the populations, especially when the age structure of birds taken as bycatch is unknown. In the extreme case that they were all breeders from the Norwegian colonies, it would have decreased annual survival by 0.4 percentage points (1300 of 300,000), reducing the long-term survival rate from 93.1% to the observed 92.7% [42] and accounting for approximately 6% of the annual loss of adults. Empirical data on the age distribution and origins of the birds affected would most likely reduce this estimate significantly. The inclusion of extreme events in the estimates would have the opposite effect.

An obvious restriction in our study is the inability to estimate and predict where, how and when extreme bycatch events might occur, despite having a rather large dataset. We decided to exclude the extreme events when constructing the GLMM-models, as they were very rare in our data and could thus not be predicted with any statistical reliability. Given that the three extreme events represent less than 0.02% of the data, the episodes most likely reflect rare peaks in the spatial-temporal distribution overlap between birds and fishing activity in an area over a short period, for instance when the seabirds and the fish are targeting high concentrations of the same prey. Up-scaled estimates based on a few such events should in general be handled with caution, as they are likely to cause much controversy and be highly biased, such as the extreme estimates reported by Strann et al. [43]. On the other hand, the rarity of these extreme events in the reference fleet might not reflect the true situation for the whole fleet. Even though we have confidence that the fishers in the reference fleet report true bycatch numbers due to the close follow up by IMR, they might also be more focused on avoiding hot spots and situations producing extreme bycatch events. However, we believe this is also the situation for most fishers, as getting a large seabird bycatch is in general an unwanted situation. The unpredictable occurrence of such extreme events, and in particular how they might modify and/or bias bycatch estimates, emphasizes the need for either more and longer time series, or carefully designed bycatch studies to better capture the real frequency and implications of such events. Our results indicate in particular that sampling effort should be increased in areas where the fishery targets fish species in shallow, nearshore waters where they coincide with high densities of diving seabirds. It is also informative to present estimates both with and without extreme and rare bycatch events to better understand how these events alter the estimates.

For reasons discussed above, the population effects of the Norwegian gillnet fishery on seabirds are somewhat inconclusive, but previous work has raised substantial conservation concerns related to the level of bycatch of diving species in gillnets [1, 6] and procellariform species in longline fisheries (e.g., [7, 44]). Our results do, however, emphasize the need for a more holistic, spatio-temporal assessment of the effects of bycatch on a wide range of seabird species across multiple types of fisheries and fishing gear to evaluate the cumulative impact of the fishing activity. We found clear spatio-temporal trends regardless of net type used. Thus, it is likely that management would benefit from a stronger focus on general mitigation measures as discussed above, rather than a focus on fisheries defined by their target species. Also, the small-vessel gillnet fisheries obviously have broader ecological effects than seabird bycatch alone, alongside associated social benefits such as employment, economic security and food. Therefore, it is not a straightforward task to reduce bycatch by implementing what could be perceived as rather drastic mitigation measures for the fishers, such as time or area closures. Although the effect of a mitigation measure on seabird bycatch might be evident in a seabird perspective, the total outcome of the measure includes multiple aspects, as it might affect local jobs, income and food security. So, we can only agree with Northrigde et al. [32] who pointed out that management efforts need to consider all of these factors to find suitable and acceptable ways to minimize the impacts of gillnet fishing on vulnerable taxa, while maintaining, or even enhancing, the benefits these fisheries provide.

## Supporting information

S1 Equations Formulas for the mean stratified estimator and the GLMM used in the analysis. The mean stratified estimator is an estimator of bycatch per trip across all vessels. The GLMM was parameterised for both diving seabirds and surface feeding seabirds.
(DOCX)
