# Supplementary material for: Spatial and temporal variations in seabird bycatch: Incidental bycatch in the Norwegian coastal gillnet-fishery
Source: PLoS One. 2019 Mar 13;14(3):e0212786. doi: 10.1371/journal.pone.0212786 (PMC6415787; doi:10.1371/journal.pone.0212786)
Supplement: S1 Equations — The mean stratified estimator is an estimator of bycatch per trip across all vessels. The GLMM was parameterised for both diving seabirds and surface feeding seabirds. (DOCX) [file pone.0212786.s001.docx]

**Supporting information**

**S1 Equations**

**Formulas for the mean stratified estimator**

For trip $k$by a vessel in stratum $h$ let $X_{i, k,h}$ be the bycatch of seabirds in numbers.

An estimator for mean bycatch per trip across all vessels in $h$ is then

$\bar{x}_{h}=\frac{\sum_{i} \sum_{k} \hat{x}_{i,k,h}}{n_{h}}$ (1.1)

where $n_{h}$ is the total number of trips in area$h$. An estimator for the total bycatch in $h$

$\hat{X}_{h}=N_{h}\bar{x}_{h}$ (1.2)

with variance

$var (\hat{X}_{h})=N_{h}^{2}var (\bar{x}_{h})$ (1.3)

where$N_{h}$ is the total number of trips in $h$ by all vessels.

**General linear mixed effect model**

In a general notion, seabird bycatch (Y) on trip *j* at vessel *i* was modelled according to:

$$Y_{ij} \sim NB(\mu_{ij}, k)$$

$E(Y_{ij}$) = $\mu_{ij}$ and $var\left( Y_{ij} \right)= \mu_{ij}+ \frac{\mu_{ij}^{2}}{k}$

$${log(\mu}_{ij})=\alpha+ \beta_{1ij}x_{1ij}+\ldots+\beta_{nij}x_{nij}+b_{i}+ \varepsilon_{ij}$$

where $\alpha$ and βs represent coefficients under estimation, x_1_-x_n_ represent candidate variables as described in Table 3, $b_{i}$ is the random vessel-specific intercept (assumed to be independent and identically distributed as $N(0,\sigma_{Vessel}^{2})$), and $\varepsilon_{ij}$ is the random residual variation.
